# 3D-printed micro bubble column reactor with integrated microsensors for biotechnological applications: From design to evaluation

**DOI:** 10.1038/s41598-021-86654-9

**Published:** 2021-03-31

**Authors:** Lasse Jannis Frey, David Vorländer, Hendrik Ostsieker, Detlev Rasch, Jan-Luca Lohse, Maximilian Breitfeld, Jan-Hendrik Grosch, Gregor D. Wehinger, Janina Bahnemann, Rainer Krull

**Affiliations:** 1 Institute of Biochemical Engineering, Braunschweig University of Technology, Rebenring 56, 38106 Braunschweig, Germany; 2 Center of Pharmaceutical Engineering, Braunschweig University of Technology, Franz-Liszt-Str. 35a, 38106 Braunschweig, Germany; 3 Institute of Technical Chemistry, Leibniz University Hannover, Callinstr. 5, 30167 Hannover, Germany; 4 Institute of Chemical and Electrochemical Process Engineering, Clausthal University of Technology, Leibnizstr. 17, 38678 Clausthal-Zellerfeld, Germany

**Keywords:** Biotechnology, Engineering, Materials science

## Abstract

With the technological advances in 3D printing technology, which are associated with ever-increasing printing resolution, additive manufacturing is now increasingly being used for rapid manufacturing of complex devices including microsystems development for laboratory applications. Personalized experimental devices or entire bioreactors of high complexity can be manufactured within few hours from start to finish. This study presents a customized 3D-printed micro bubble column reactor (3D-µBCR), which can be used for the cultivation of microorganisms (e.g., *Saccharomyces cerevisiae*) and allows online-monitoring of process parameters through integrated microsensor technology. The modular 3D-µBCR achieves rapid homogenization in less than 1 s and high oxygen transfer with *k*_L_*a* values up to 788 h^−1^ and is able to monitor biomass, pH, and DOT in the fluid phase, as well as CO_2_ and O_2_ in the gas phase. By extensive comparison of different reactor designs, the influence of the geometry on the resulting hydrodynamics was investigated. In order to quantify local flow patterns in the fluid, a three-dimensional and transient multiphase *Computational Fluid Dynamics* model was successfully developed and applied. The presented 3D-µBCR shows enormous potential for experimental parallelization and enables a high level of flexibility in reactor design, which can support versatile process development.

## Introduction

For biopharmaceutical process development, microbioreactors (MBRs) with a cultivation volume ≤ 1000 µL play an essential role—especially for screening new production strains and/or process optimization. Various MBR systems have been developed for automated and parallel operation to enable, e.g., a realistic scale up/down of biotechnological processes^1^, characterization of mammalian cell heterogeneity^2^, and the screening of whole cell and biotransformation systems^3^. This MBR technology allows researchers to obtain quantitative data concerning the most important process variables from a large number of simultaneously running cultivation approaches in real-time, with both high data density and accuracy^4–6^. For example, Edlich et al.^7^ developed a 10 µL horizontal flow and passively gassed MBR system made of glass and PDMS with integrated online sensors for optical density (OD) and dissolved oxygen tension (DOT), which was used for the cultivation of *Saccharomyces cerevisiae (S. cerevisiae)*. A further MBR system resulted in a vertically operated, multi-phase bubble column microreactor, which allows an improved degassing of the cultivation medium^8,9^. Lladó Maldonado et al.^10^ manufactured a 60 µL microbubble column reactor (µBC) entirely from borosilicate glass (since the use of PDMS is only conditionally suitable for the production and use of MBR systems). The µBC was numerically simulated with respect to its mass transfer and mixing behaviour by means of *Computational Fluid Dynamics* (CFD) modelling, and was satisfactorily validated with experimental data. A cuvette MBR with online sensor technology for pH, OD, DOT, and glucose measurements was also recently developed^11^. The functionality and the high potential of the cuvette MBR has been demonstrated for cultivation in batch mode with *S. cerevisiae*^11^ and chemostat mode with *Staphyllococcus carnosus*^12^. Importantly, however, all of these systems are prototypes developed specifically for use in their respective applications. Accordingly, transferring the underlying technology to other cell systems or process modes, still typically requires a significant adaptation of the MBR design—which often necessitates complex manufacturing steps.

Additive manufacturing (also known as 3D printing) has become a focus of interest for a variety of biotechnological applications in recent years. A major advantage of 3D-printed microsystems over traditional manufacturing methods is that desired prototypes can be produced within just a few hours of the completion of design, in high-resolution structures in the range of a few micrometers. This facilitates system optimization to be implemented and tested quickly. Microfluidic systems are increasingly being manufactured using 3D printing^13–17^. Krujatz et al.^18,19^ developed and manufactured a flat-panel airlift (FPA) reactor with a reaction volume of 15 mL using MicroLED technology in 3D printing, and equipped it with sensors and real-time measurement technology (including LED light sources for homogeneous light distribution, OD, pH, DOT and pCO_2_). The miniaturized FPA photobioreactor was characterized with respect to the essential fluid-dynamic process parameters of mixing time, gas content, and *k*_L_*a* value. Panjan et al.^20^ presented an approach of a 3D-printed 1000 µL MBR for the cultivation of *S. cerevisiae*, and yet another application of 3D-printed MBR for the cultivation of microalgae was described by Cox^21^. In principle, these studies show the great potential of 3D printing for the fabrication of MBR systems.

This study presents for the first time a novel, modular design and reproducible setup of a 3D-printed micro bubble column reactor (3D-µBCR) with integrated sensor technology and a cultivation volume of 550 µL. The characterization of mixing time and oxygen transfer, as well as the evaluation of the 3D-µBCR are demonstrated for the cultivation of *S. cerevisiae* CCOS 538 as a model system. In order to ensure efficient process analytics, the 3D-µBCR is equipped with non-invasive online sensors to monitor temperature, pH, DOT, and O_2_ and CO_2_ in the gas phase. Without affecting the liquid level this comprehensive set of online analytics allows for a precise process control. Due to the small reaction volume, sampling and offline analysis related thereto is only possible to a limited extend.

The development of the 3D-µBCR using rapid prototyping process efficiently facilitates optimization of the reactor design for customized setups. The fluid dynamics are also characterized with multiphase 3D transient CFD simulations. This provides a suitable complementary tool for iterative improvement for the optimization of the 3D-µBCR-system, so that it can be adapted and tested in the shortest amount of time.

## Results and discussion

### Design of the 3D-printed micro bubble column reactor

In order to induce active mixing and enhance mass transfer inside the cultivation chamber, the MBR is designed as a bubble column rector, which induces pressurized air at the reactor bottom via a nozzle with 0.3 mm diameter. Hereafter, it is therefore referred to as 3D-printed micro bubble column reactor (3D-µBCR). The momentum of the rising gas bubbles is transferred into the fluid, preventing concentration and temperature gradients. The increased specific surface area of the dispersed gas also enhances mass transfer from the gas to the liquid phase. Both the design principle of the 3D-µBCR, as well as the shape and dimensions of the cultivation chamber were adapted from Lladó Maldonado et al.^10^.

**Figure 1:**
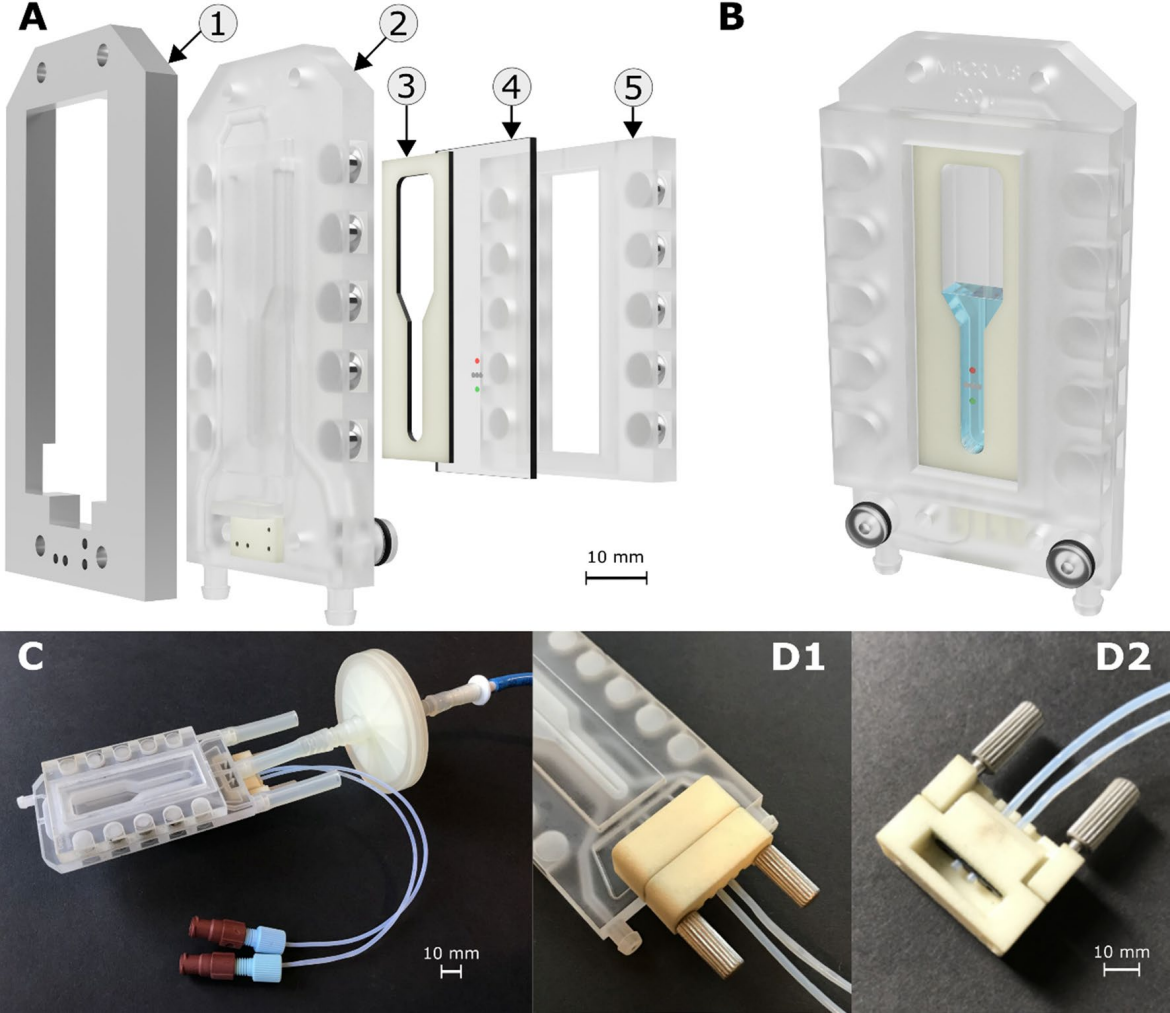
3D-printed micro bubble column reactor (3D-µBCR). (**A**) Exploded view of reactor components with (1) connector clip with fluid inlets, (2) 3D-µBCR (reactor module) with magnets, (3) silicone sealing, (4) sensor plate with inkjet-printed sensor spots in front side, (5) frame for sensor plate with magnets. The sensor plate (4) inside its frame (5) encloses together with the silicone sealing (3) the reaction volume inside the reactor module (2). These parts are magnetically kept together. For leak prove sealing, the connector clip applies pressure by clamping all parts together using screws. On the sensor plate (4) the microsensors are spotted to be in contact with the reaction fluid. (**B**) Assembled 3D-µBCR in rendered presentation (rear view). (**C**) Image of fully assembled 3D-µBCR with fluid connectors, tubing and inlet filter. (**D**1, **D**2) 3D-printed fluid connector for stable fluid supply.

The 3D-µBCR is modularly constructed and assembled out of five main components which are illustrated in Fig. 1A. The connector clip with fluid inlets is shown in (1), the actual cultivation chamber is defined by the reactor module (2), whose reverse side is covered by a sensor plate (4) embedded in a frame (5). The reactor module and sensor plate framing are bound together via ten neodymium magnets (6 mm × 3 mm, Magnetastico, Karlsfeld, Germany) attached to each part, which facilitate quick assembly. To avoid any leakage, a silicone sealing (3) is also interlaid in between the reactor module (2) and the sensor plate (4). The assembled 3D-µBCR module (Fig. 1B, C) is then mounted to a connector clip, with fluid inlets (1) that allow for the feeding of process fluids as well as the induction of gassing. The 3D-µBCR features four inlet channels with an inner diameter of 0.8 mm—three of which lead to the bottom of the reactor, and one to the upper area of the reaction fluid. A leakage-proof connection between the tube and the 3D-µBCR is further guaranteed by a fluid connector (see Fig. 1D). The reactor module and the sensor plate frame are both manufactured via 3D printing.

The 3D-printed parts of the 3D-µBCR are intended as a disposable system. However, the cover plate with the printed sensor spots is intended to be a recyclable system. Due to the modular design of the 3D-printed reactor, the sensor plate can be removed and reinstalled quite easily. However, previous work has shown that the 3D-printed material is also suitable for repeated experiments and biological applications and can be reused (e.g., Arshavsky-Graham et al.^22^). The material can also be sterilized. However, long-term studies on material stability do not yet exist.

### Variation of reactor geometry

A physical description of processes observed in bioreactors can be analyzed and quantified using specific parameters such as the mixing time (*t*_M_) and the volumetric oxygen transfer coefficient (*k*_L_*a*). In bubble column reactors, these parameters are primarily affected by the geometrical relations and the volumetric gas flow rate introduced into the fluid. The resulting superficial gas velocity *u*_g_ can be calculated using Eq. 1, dividing the volumetric gas flow rate $\dot{V}_{g}$ by the reactor cross section *A*.

1 $$u_{{\text{g}}} = \frac{{\dot{V}_{{\text{g}}} }}{A}$$

**Figure 2:**
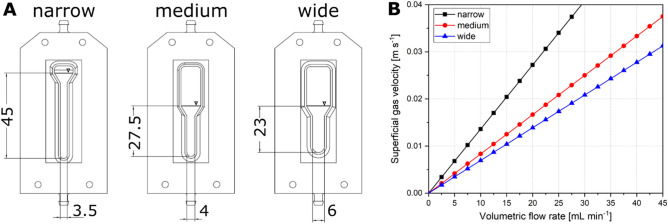
(**A**) Technical drawings of investigated 3D-µBCR geometries resulting in a similar volume of 550 µL. All dimensions are given in millimeters. The reactor geometries are designed with a rectangular cross section with dimensions listed in Table 1. (**B**) Superficial gas velocity in dependence on the volumetric flow rate for the three different reactor geometries calculated using Eq. 1.

Rapid prototyping, in combination with the modular construction of the 3D-µBCR, enables a high level of flexibility in reactor development. Both the shape and the geometry of the cultivation chamber can be altered without changing the surrounding reactor. This feature was used to design three different reactor geometries and to investigate the effect on hydrodynamic parameters inside the reaction chamber (Fig. 2A).

Even as the reactor volume was kept constant, however, the height and width of the reaction chamber were altered—resulting in a narrow, medium, or wide geometry, respectively. The depth of the cultivation chambers in these different configurations is 3.5, 5, and 4 mm, and each chamber results in a different liquid level. The dimensions of the three µBCR geometries with resulting reactor cross sectional area are shown in Table 1.

**Table 1:** Dimensions of evaluated reactor geometries with respective reactor cross sections and liquid levels.

Reactor geometry	Width [mm]	Depth [mm]	Height [mm]	Reactor cross sectional area *A* [mm^2^]	Height/diameter ratio ^a^	Width headspace [mm]
Narrow	3.5	3.5	45	12.25	11.4	9.5
Medium	4	5	27.5	20	5.4	10
Wide	6	4	23	24	4.2	12

^a^For the height/diameter ratio a circular area of similar *A* was assumed. The following diameters were used: narrow: *d* = 3.95 mm; medium: *d* = 5.05 mm; wide: *d* = 5.53 mm.

Using the reactor cross sectional area *A*, the superficial gas velocity can be calculated for each reactor geometry depending on the volumetric flow rate (see Eq. 1). For a volumetric flow rate of up to 45 mL min^−1^ a maximum superficial gas velocity of 0.061 m s^−1^ can be achieved (Fig. 2B). Consequently, a larger reactor cross sectional area leads to decreased superficial gas velocities. The superficial gas velocity is directly linked to the hydrodynamic characteristics inside the reaction chamber.

### Characterization of reactor geometries

**Figure 3:**
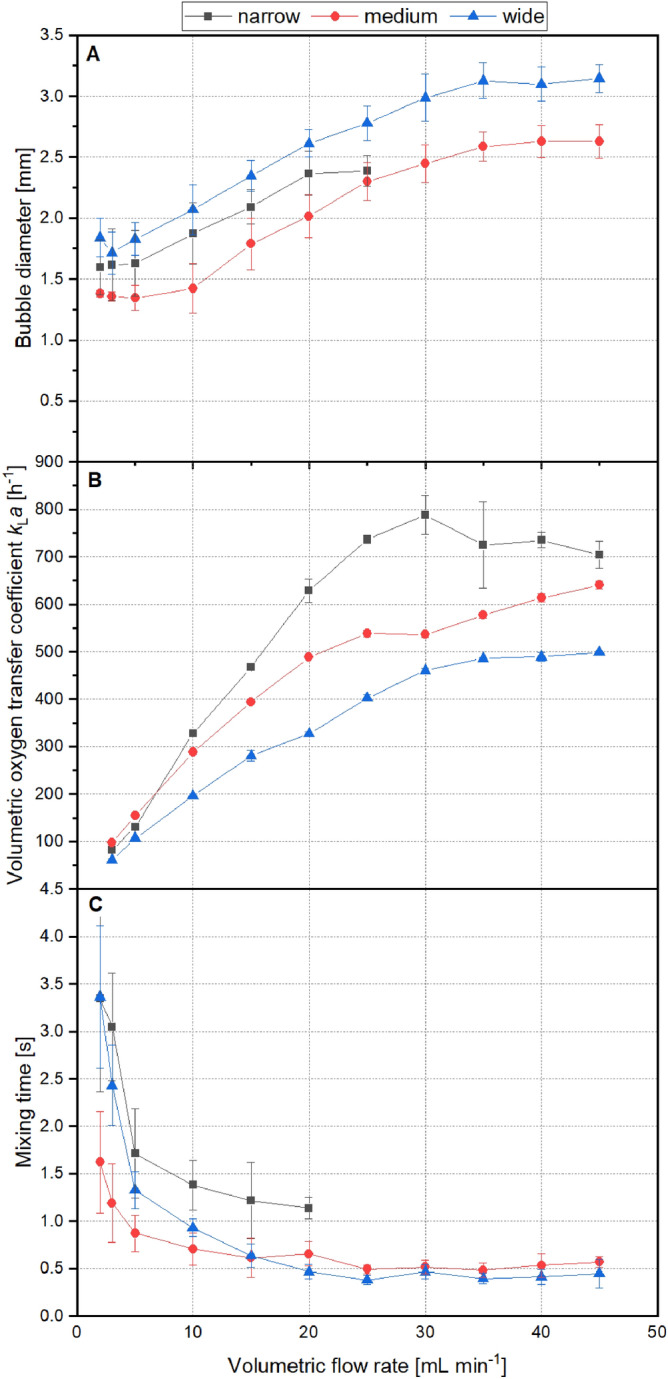
Characterization of 3D-µBCR geometries depending on volumetric flow rate. (**A**) Bubble diameter depending on the gas flow rate for different reactor geometries. Diameters were determined using image analysis (n < 400). (**B**) Volumetric oxygen transfer coefficient *k*_L_*a* depending on the gas flow rate for the different reactor geometries. (A) + (B) All data is shown in mean values of triplicates with standard deviation. (**C**) Analysis of mixing time *t*_M_ depending on the volumetric flow rate for the different reactor geometries.

To evaluate the influence of the reactor geometry on the resulting hydrodynamics, key parameters for the mass transfer in 3D-µBCRs were investigated. One decisive factor is the equivalent bubble diameter *d*_e_ of the gas introduced into the 3D-µBCR rising through the liquid phase while promoting fluid flow and mass transfer in the latter. *d*_e_ has a significant influence on *k*_L_*a*, due to the varied phase boundary interface. *d*_e_ was therefore determined for all three reactor geometries depending on the volumetric flow rate (Fig. 3A).

Bubble diameters between 1.3 and 3.1 mm were detected. A higher volumetric flow rate generally leads to an increase in bubble diameter. Additionally, the reactor geometry apparently has an influence on *d*_e_. In the widest reactor, bubbles up to 3.2 mm were formed, followed by the medium reactor (2.6 mm). In the narrow geometry, however, a maximum bubble diameter of 2.4 mm was detected at 25 mL min^−1^. For higher flow rates in the narrow geometry, bubbles were stabilized on the reactor walls leading to a coalescence of bubbles. As a consequence, the fluid was conveyed upwards and spilled out of the reactor volume. Both medium and wide geometry reach maximum *d*_e_ at 35 mL min^−1^. Higher volumetric flow rates do not result in larger *d*_e_.

Aside from bubble diameter, the effect of the reactor geometry on the resulting *k*_L_*a* was investigated for volumetric flow rates between 2 and 45 mL min^−1^ (Fig. 3B). Comparable to *d*_e_, the *k*_L_*a* rises with higher volumetric flow rates for all reactor geometries. For a given flow rate, the wide geometry generally results in lower *k*_L_*a* values. Here, *k*_L_*a* values between 62 and 499 h^−1^ were monitored. The medium geometry gives *k*_L_*a* values between 97 and 640 h^−1^ and the narrow geometry results in *k*_L_*a* values between 82 and 788 h^−1^. The largest value in the narrow geometry was achieved for a flow rate of 30 mL min^−1^. An additional increase of the flow rate did not result in higher *k*_L_*a* values.

Consequently, oxygen transfer can be affected by both the reactor geometry and the volumetric flow rate. The *k*_L_*a* dependency on the reactor geometry can be explained by the increased height of the fluid, leading to higher residence times for a lower reactor cross sectional area. Put differently, in a narrow reactor geometry, the duration for mass transfer is enhanced. Additionally, higher residence times interact with lower *d*_e_ providing a higher phase boundary interface for a given volumetric flow rate.

Compared to other *k*_L_*a* values reported in the literature, considerably high mass transfer coefficients can be achieved in the 3D-µBCR discussed in this study. Peterat et al.^9^ reported on *k*_L_*a* values up to 470 h^−1^, but within a significantly smaller system of 70 µL fluid volume. Lladó Maldonado achieved *k*_L_*a* values up to 204 h^−1^. Higher *k*_L_*a* values were also reported by Kheradmandnia et al.^23^ for a 20 mL miniaturized bubble column with an oxygen transfer of 800 h^−1^. Lab-scale stirred tank bioreactors were reported to have *k*_L_*a* values up to 400 h^−1^, shake flasks were characterized with up to 180 h^−1^ and micro titer plates up to 250 h^−1^^24–26^. Conclusively, the cultivation broth in the 3D-µBCR discussed in this study can be sufficiently fed with oxygen (through active pneumatic gassing) to open up a wide operating window for various biotechnological applications.

In addition to the oxygen supply for aerobic cultivations, one main task of a bioreactor is the rapid homogenization of the cultivation broth—to avoid temperature and concentration gradients, as well as to counteract sedimentation of cells^5^. Quantification of homogenization and mixing performance can be achieved by calculating the mixing time *t*_M_, which is defined here as the time required to achieve 95% homogeneity^27^. *t*_M_ was determined for all reactor geometries using a colorimetric method combined with subsequent image analysis (Fig. 3C).

*t*_M_ decreased in all reactor geometries as volumetric flow rates rose. For flow rates up to 15 mL min^−1^, the medium geometry showed the lowest *t*_M,_ followed by the wider geometry and then the narrower one. In the narrow geometry, flow rates above 20 mL min^−1^ led to a reactor blow out, which was previously reported for the bubble diameter. Hence, minimal *t*_M_ was determined at 20 mL min^−1^ with 1.1 s. In both the medium and the wide geometry, a minimal *t*_M_ of 0.5 s was determined—which can be considered to constitute rapid homogenization.

The injected gas and the rising bubbles imply a momentum exchange with the surrounding fluid, which causes a fluid flow and promotes homogenization. Larger *t*_M_ at lower flow rates can be explained by the fact that single gas bubbles fail to evoke mixing in the entire reaction fluid volume. If the volumetric flow rate is enhanced, the frequency of rising bubbles is increased, eventually forming chains of rising bubbles. This term is used if the wake of single bubbles mutually influences each other causing a flow profile in the reaction fluid^28^. In the narrow reactor geometry, higher *t*_M_ can stem from the lack of an overall flow profile. Due to the lower cross-sectional area, the influence of wall effects increases causing a deceleration of the fluid flow. However, the mixing time-profiles of all geometries exhibit a strong similarity, showing only minor differences for a given volumetric flow rate. *t*_M_ is primarily affected by the volumetric flow rate, while the reactor geometry is only playing a limited role.

In comparison to most actively gassed MBR-systems reported in literature, the present 3D-µBCR-system achieved faster homogenization. Peterat et al.^9^ have reported a minimum mixing time of 1.4 s in a 70 µL reaction volume. Lladó Maldonado et al.^10^ achieved a minimum mixing time of 1 s in 500 µL. Direct comparison is complicated by the different tracer injection methods and lower volumetric flow rates applied by Peterat^29^ and Lladó Maldonado et al.^10^, however. In other stirred miniaturized bioreactors, like the Ambr15-system (Sartorius, Göttingen, Germany), mixing times between 5 and 20 s have been reported^30^. Avoiding movable mixing parts inside the reaction chamber, the present 3D-µBCR-systems exceed this mixing performance.

In conclusion, the present 3D-µBCR-systems achieve rapid homogenization in less than 1 s and high oxygen transfer with *k*_L_*a* values up to 788 h^−1^. Due to the lowest *t*_M_, the smallest bubble diameter, and sufficiently large *k*_L_*a* values, the medium reactor geometry (*h/d* ratio 5.4; compare Fig. 2A) proved to be particularly advantageous for biotechnological applications enabling flexible process development.

### Analysis of fluid dynamics

**Figure 4:**
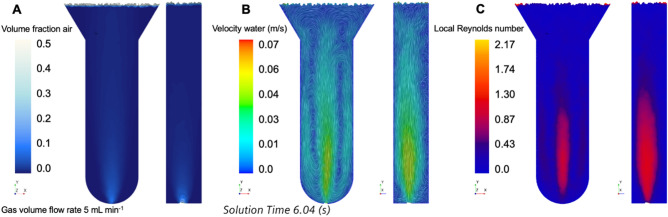
(**A**) Volume fraction (VF) of air, (**B**) velocity magnitude of water, and (**C**) local Reynolds number. Gas volume flow rate 5 mL min^−1^. (**A**) and (**C**) are time-averaged over 6 s.

**Figure 5:**
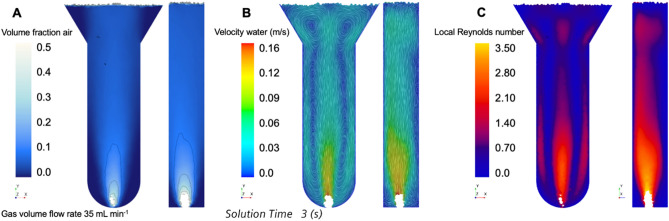
(**A**) Volume fraction of air, (**B**) velocity magnitude of water, and (**C**) local Reynolds number. Gas volume flow rate 35 mL min^−1^. (**A**) and (**B**) are time-averaged over 3 s.

In order to quantify local flow patterns in the fluid, a three-dimensional and transient multiphase *computation fluid dynamics* (CFD) model was developed. Figure 4A and Fig. 5A present results from the multiphase 3D transient CFD simulations. The volume fraction of air is shown on a midplane and side view inside the fluid phase of the column for a gas volume flow rate of 5 and 35 mL min^−1^ and averaged over 3 and 6 s, respectively. The iso-lines have a spacing of 0.1 volume fraction of air. Pure air is injected at the bottom of the column and then rises up towards the top of the fluid phase. At the low gas volume flow rate, a small amount of air is dispersed into the water phase. The time-averaged volume fraction of air is recognizable only at very bottom of the column, just above the inlet. With increasing distance from the inlet, the volume fraction of air decreases to small values that rapidly approach zero. The larger the gas volume flow rate, the larger the gas hold-up—hence the larger the volume fraction of air in the fluid phase. This can be seen for $\dot{V}$ = 35 mL min^−1^ in Fig. 5A, where the volume fraction of air is significantly larger than zero in many regions of the column. The upper sides and lower side regions are characterized by a very small volume fraction of air, however. This becomes clearer when the water velocity vector fields in Fig. 4B and Fig. 5B are considered, that are shown here at a specific time step (see details in the figures: solution time). The injected air pushes the water phase to the top, inducing a jet-like situation at the bottom of the column. Axisymmetric and downward-facing flows occur close to the side walls in the *xy*-plane, and because the air inlet is not exactly in the center of the bottom, a non-symmetric flow field develops along the *yz*-plane. At the top, two additional distinct eddies also occur. In Fig. 4C and Fig. 5C, the time-averaged local Reynolds number is shown taking the local liquid velocity (continuous phase) into account (Eq. 2)^10^:

2 $$Re_{L} \equiv \frac{{\rho_{{\text{c}}} \cdot d_{{\text{h}}} \cdot \overline{{\left| {{\mathbf{v}}_{{\text{c}}} } \right|}} }}{{\mu_{{\text{c}}} }}$$

*ρ*_c_ is the liquid (continuous) density, *μ*_c_ is the liquid dynamic viscosity, *d*_h_ is the hydraulic diameter of the cross section of the column, and $\overline{{\left| {{\mathbf{v}}_{{\text{c}}} } \right|}}$ is the time-averaged velocity magnitude of the liquid phase. This local Reynolds number can be determined in every cell in the computational domain, and is therefore different to the Reynolds number formulated with the superficial liquid velocity. The local Reynolds number for the low air volume flow rate is in the range between zero and 1.75, whereas the largest numbers occur in the lower central region.

A different pattern emerges for the larger volume flow rate of air. The lower central region again shows high Reynolds numbers, and the eddies close to the lower side walls also enlarge with increasing *Re*_L_. In addition, the two eddies at the top of the liquid phase accelerate the liquid, which yields in zones of larger *Re*_L_ in the upper center and at the edges of the column. The side view of Fig. 5C reveals that close to the back side of the column, a region of stagnant water forms. For this large volume gas flow rate, the mean liquid Reynolds numbers are in the range between zero and 3.5.

**Figure 6:**
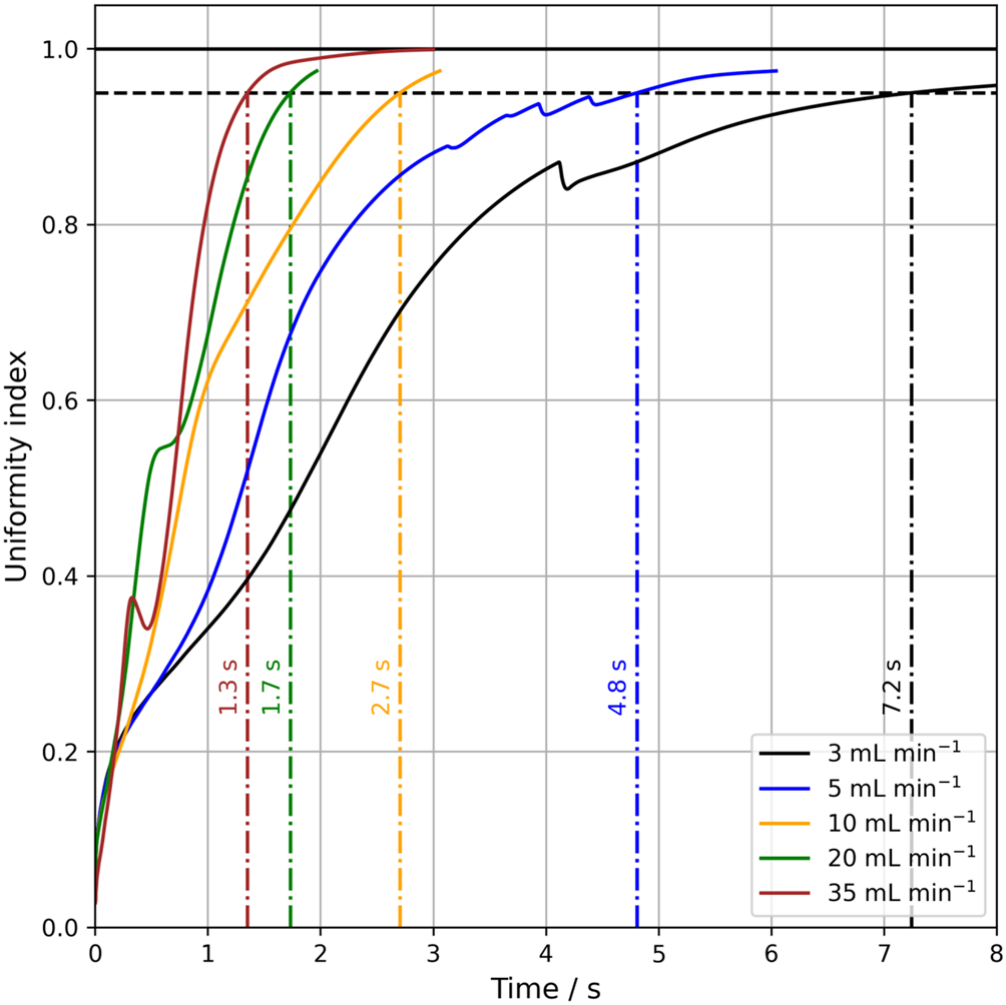
Uniformity index over time from CFD simulations for different volume flow rates.

Figure 6 shows the temporal development of the uniformity index (i.e., the degree of homogeneity) for the different gas volume flow rates for the CFD simulation results. The mixing time represents the situation where this index reaches 95%^27^. In general, the mixing time decreases with increasing gas volume flow rates from 1.3 to 7.2 s for 3 and 35 mL min^−1^, respectively. Whereas the general trend of the experiments is well captured with the CFD model, it is important to note that the exact mixing time is overestimated by a factor of approximately 2.5. The experimental procedure of the mixing time measurements and consecutive image analysis might explain this discrepancy. The experimental injection of a tracer (i.e., a colored liquid phase) introduces an additional convective flow which is not taken into account in the CFD simulations. Furthermore, the applied drag models in the Eulerian multiphase (EMP) framework were developed for large bubble columns. The interplay between bubbles, liquid, and confining walls might differ significantly between this micro bubble column and the typically investigated industrial scale columns. As a consequence, a fundamental investigation of bubble induced flows within micro bubble columns is needed to apply EMP sub-models in the CFD framework. In addition, bubble-induced turbulence (i.e., turbulence generated by interactions of bubble wakes) was not taken into account. Alméras et al.^31^ have recently presented a modeling approach in which the transport of a species can be modeled by an effective diffusion in a complex bubbly flow at moderate gas volume fraction. Further investigations of shear and bubble induced turbulence in micro bubble reactors are warranted.

### Biological application: cultivation of *Saccharomyces cerevisiae* CCOS 538

Subsequently, cultivations of *S. cerevisiae* CCOS 538 were performed as a proof-of-concept proving the applicability of the 3D-µBCR-system. The hydrodynamic characterization of the 3D-µBCR has shown rapid homogenization and adequate oxygen supply within the fluid phase that exceeds that seen in other systems reported in the literature. The modular design of the present system also offers greater flexibility and suitable sensor integration compared to previously published MBR-systems^4,32,33^. To demonstrate the performance of the 3D-µBCR for microbial organisms and allow for comparison to other MBR-systems, the medium reactor geometry was used to cultivate the aerobic yeast *S. cerevisiae* CCOS 538 in submerse batch mode. This strain has previously been investigated in detail and reported in the literature^11^. Additionally, due to its diauxotrophic growth, it is particularly well suited as a model organism to validate a cultivation system with its implemented sensors allowing for monitoring of different metabolic phases.

**Figure 7:**
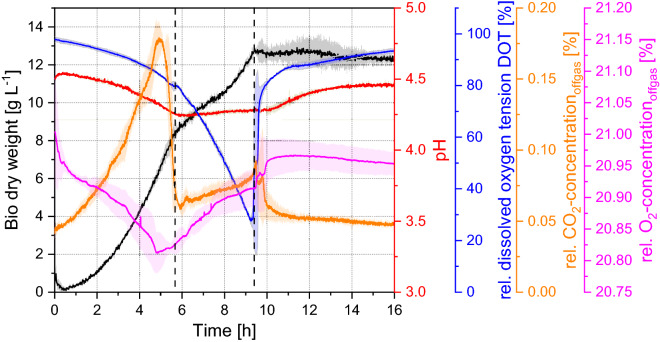
Cultivation of *Saccharomyces cerevisiae* CCOS 538 in modified Verduyn medium containing 20 g L^−1^ glucose at 30 °C at a superficial gas velocity of 0.0125 m s^−1^ (volumetric flow rate of 15 mL min^−1^). Black: Concentration of bio dry weight; Red: pH; Blue: Relative dissolved oxygen tension; Orange: Relative CO_2_ concentration in the off-gas; Pink: Relative oxygen concentration in the off-gas. Data are given in mean values of duplicates with standard deviation.

The present 3D-µBCR is able to monitor biomass, pH, and DOT online in the fluid phase, as well as the concentration of CO_2_ and O_2_ in the off-gas. Figure 7 shows the course of cultivations in duplicate of *S. cerevisiae* CCOS 538 in the 3D-µBCR applying a volumetric flow rate of 15 mL min^−1^. All data was generated using the implemented online analytics described in the subsequent materials and methods section. Hence, the liquid level was not affected by withdrawing offline samples.

In general, both cultivations exhibit a close match, with only slight deviations. Accordingly, the mean values of each parameter curve are used in the following to determine key cultivation parameters. Looking at the bio dry weight (BDW) curve, two distinct growth phases become evidence: First, the initial growth phase, starting after a lag phase of 0.5 h, shows an exponential increase until 6 h of cultivation with a maximum growth rate of 0.403 ± 0.02 h^−1^. This growth is assumed to be based on glucose consumption as primary energy source, which is very probably consumed after 5.3 h, since the CO_2_ concentration in the off-gas significantly drops. To this point, approximately 6.3 g L^−1^ BDW were produced, resulting in a glucose yield coefficient *Y*_X/S_ of 0.3 g_BDW_ g_glucose_^−1^ for the given initial glucose concentration of 20 g L^−1^. In future investigations, glucose analytics can elucidate this assumption. Second, the second growth phase between 8 and 9.8 h appears to be linear and displays a significantly smaller growth rate of 0.147 ± 0.01 h^−1^. Here, growth is assumed to be based on ethanol, which is depleted after 10 h. Thereafter, the cultivation enters a stationary phase which results in a maximum BDW of 12.1 g L^−1^ and a total yield coefficient of *Y*_X/S_ of 0.61 g_BDW_ g_glucose_^−1^.

The diauxic growth is additionally illustrated in the course of the CO_2_ and O_2_ concentrations. During glucose-based growth, CO_2_ concentration in the off-gas increases in proportion to the BDW and is accompanied by a decrease of the O_2_ concentration in the off-gas. In the second growth phase, CO_2_ increases in the off-gas due to the metabolization of the carbon source. The DOT in the liquid phase constantly decreases during both growth phases. In the off-gas, only a decrease during the first phase was monitored. The course of the DOT concentrations can be explained by the glucose metabolism under the *Crabtree*-effect and the ethanol consumption. The demand for oxygen during glucose-based growth is lower compared to ethanol-based growth^34,35^. For each mol glucose consumed in cell metabolism, one mol ethanol and 2.16 mol of CO_2_ is formed. During considerably smaller growth based on ethanol, the demand for oxygen is constantly rising, whereas less CO_2_ is being formed. Hence, another decrease of O_2_ in the off-gas was expected during the second growth phase. This effect should be clarified in future investigations, what can be supported by the implementation of additional online sensors, such as glucose and ethanol, allowing for a broader monitoring of all biological processes.

The pH value shows good concordance to the cultivations performed by Lladó Maldonado et al.^10^. It decreases from initially pH = 4.5 to 4.3 during glucose-based growth; remains constant during second growth phase; and then increases again during stationary growth. This development can be explained by the formation of organic acids during glucose metabolism. During ethanol-based growth, formation of organic acids is decreased. Once glucose and ethanol are depleted, *S. cerevisiae* uses organic acids as an energy source^36^.

The potential for rapidly generating data at a lower reaction volume through the present 3D-µBCR is evident. Paalme et al.^37^ reported on specific growth rates *µ*_glucose_ and *µ*_ethanol_ of 0.44 and 0.13 h^− 1^ respectively for a 2.5 L bioreactor—but with only 2.5 g L^−1^ initial glucose concentration. Additionally, Kuhlmann et al.^38^ reported on specific growth rates *µ*_glucose_ and *µ*_ethanol_ of 0.40 and 0.13 h^−1^ respectively for a 2.5 L bioreactor with 30 g L^−1^ initial glucose concentration. Hence, the presented 3D-µBCR is capable of generating experimental data in 550 µL comparable to lab-scale bioreactor with a 4500 × higher reaction volume. Low standard deviations underline robust experimental conditions generated by the 3D-µBCR for identical cultivations performed in duplicate, proving the applicability of the MBR-system. For quantitative analysis, however, a distinct focus on batch-to batch deviations has to be set.

## Conclusions

In this study, the design, fabrication, extensive characterization, and evaluation of a novel, modular 3D-printed micro bubble column reactor (3D-µBCR) with integrated microsensors for cultivation and monitoring of microorganisms were demonstrated. The development of the 3D-µBCR using additive manufacturing efficiently facilitates optimization of the reactor design (including, e.g., 3D-printed connectors and interfaces for gas supply as well as sensor integration) for customized setups. The hydrodynamic characterization of the 3D-µBCR has shown rapid homogenization and adequate oxygen supply within the fluid phase that exceeds that seen in other systems reported in the literature. The present 3D-µBCR—with a cultivation volume of just 550 µL—has proven to successfully and reproducibly cultivate eukaryotic yeast cells (*S. cerevisiae*) and monitor important cultivation parameters using integrated microsensors. The latter enables the online monitoring of biomass, pH, and DOT in the fluid phase, as well as the concentration of CO_2_ and O_2_ in the off-gas. In this way, similar key parameters as in lab-scale bioreactors were achieved, proving the potential of the 3D-µBCR for future bioprocess development—e.g., through system parallelization and automation.

Furthermore, a three-dimensional transient multiphase CFD model was develop, with which the local flow patterns and mixing times were quantified. This model is based on the actual reactor geometry and can therefore be integrated early in the reactor design development process.

The modular design of the present system also offers greater flexibility and suitable sensor integration compared to previously published MBR-systems. The miniaturized reactor has tremendous potential for system parallelization and automation, and 3D printing provides a high degree of flexibility in reactor design, both of which can support further bioprocess development.

## Methods

### Manufacturing of micro bubble-column reactor via 3D printing

The design of the 3D-µBCR-systems was created in CAD (Fusion 360, Autodesk, Munich, Germany), and fabricated using a multijet printer (Projet MJP 2500 Plus, 3D Systems, Rock Hill, USA) by applying a layer resolution of 32 µm and a print resolution of 800 × 900 dpi. The printing platform has a size of 294 × 211 mm (with a maximum printing height of 144 mm), which allows several different parts to be produced simultaneously next to each other in one print process. VisiJet M2R-CL (containing 3-Hydroxy-2,2-dimethylpropyl-3-hydroxy-2,2-dimethyl-propionate diacrylate) was used as printing material, which was cured using UV-light and the polymerization initiator diphenyl(2,4,6-trimethylbenzoyl)phosphine oxide (3D Systems, Rock Hill, USA). For increased precision hollow cavities and channels were filled with hydroxylated wax as supporting material (VisiJet M2SUP, 3D Systems, Rock Hill, USA). In subsequent post processing, the printed parts were cooled at -18 °C for 10 min, and then incubated at 80 °C in a cleaning solution (MJP EasyClean, 3D-Systems Inc., Rock Hill, USA) for 30 min, followed by a second incubation at 70 °C in an additional cleaning agent (EZRinse-C, 3DSystems Inc., Rock Hill, USA) to remove oily residues (detailed protocol according to Siller et al.^39^). Finally, all parts were flushed with ethanol (96%, Carl Roth, Karlsruhe, Germany) for 10 min, and stored overnight in a sterile workbench to allow for evaporation of the ethanol. After irradiation with UV-light for 1 h, the 3D-µBCR was assembled under sterile conditions.

### Microbioreactor experimental setup and sensor integration

All 3D-µBCR experiments were performed in a custom made incubation chamber (45 cm × 75 cm × 45 cm) to allow for precise control of ambient temperature and humidity, as described by Peterat et al.^9^. Additionally, the temperature in the reactor was controlled using an infrared temperature sensor (MLX90614, Melexis NV, Ypern, Belgium) connected to a thermostat (Lauda Eco Silver, Lauda Dr. R. Wobser GmbH & Co., Lauda-Königshofen, Germany), pumping water through a double jacket surrounding the reaction volume of the 3D-µBCR. The temperature sensor is connected to a microcontroller (ATMega328, Arduino nano, Arduino S.r.l., Nizza, Italy) which reads the sensor and transmits the data to a computer. The latter, in turn, is connected to the internal PID controller of the thermostat. The source code for data transmission between temperature sensor and thermostat can be found in the S Supplementary information (see SI1 *Sensor controlling and read out*).

The influx of gas was pre-humidified before being introduced into the 3D-µBCR, in order to avoid evaporation of the cultivation broth (i.e., the reactor content). Precise control of the volumetric gas flow rate was obtained using a mass flow controller (El-Flow, Bronkhorst High-Tech B.V., Ruurlo, Netherlands).

**Figure 8:**
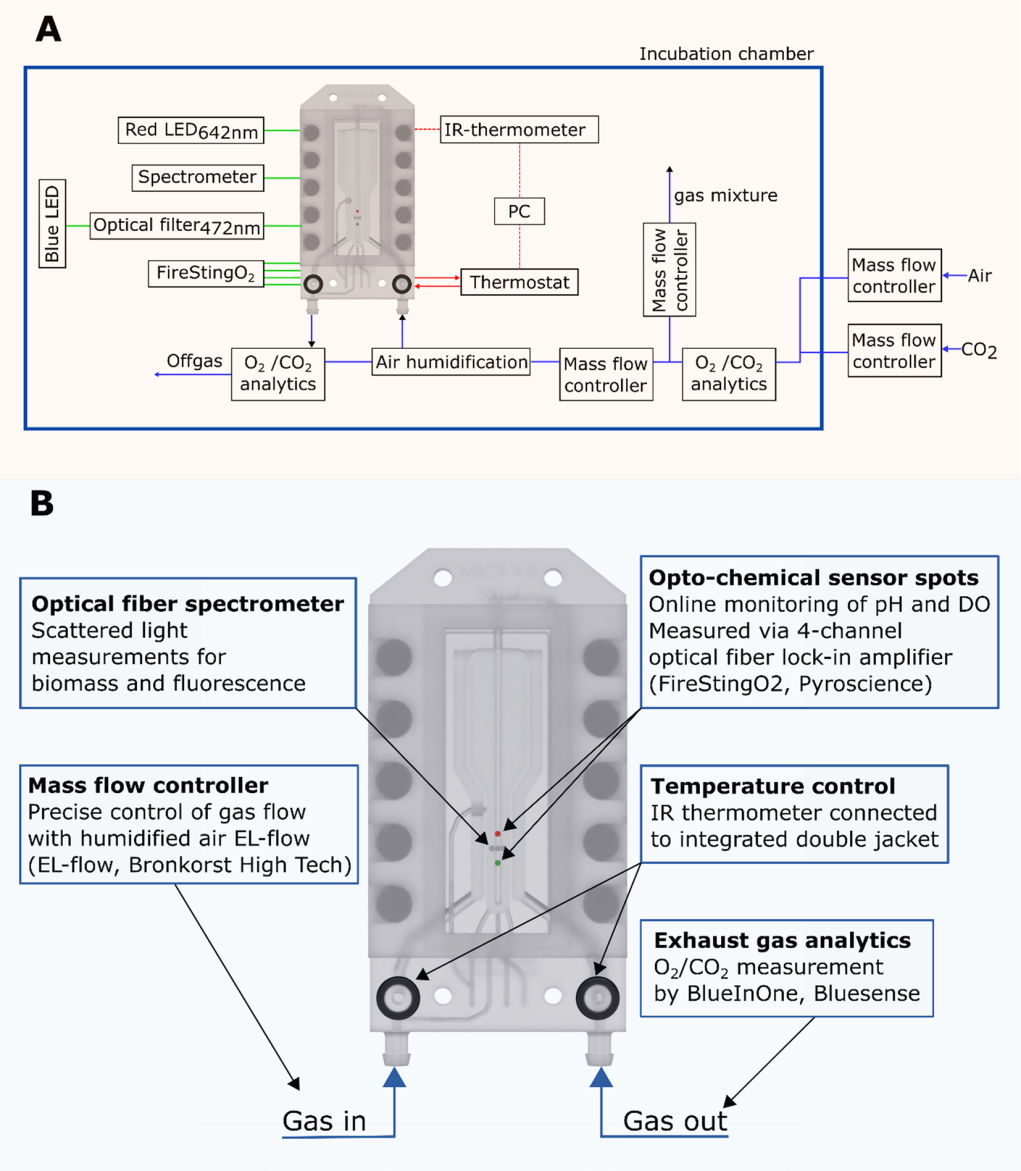
(**A**) Block diagram of the experimental setup. Optical fibers are shown in green, circulation for temperature control is shown in red and gas supply is shown in blue. (**B**) 3D-µBCR with integrated optical sensors.

The arrangement of applied online sensors is illustrated in Fig. 8. Biomass was monitored online using a scattered light (SL) sensor. An optical fiber connected to a red LED (wavelength 642 nm) was mounted at an angle of 39° to the 3D-µBCR rear panel (sensor plate) for excitation of scattered light. The latter was then read out by another optical fiber at an angle of 90° to the 3D-µBCR rear panel, which was connected to a fiber optical spectrometer (USB2000 + , Ocean Optics, Largo, USA). Scattered light intensities were measured with an integration time of 1 s and 60 data points on average, resulting in a measurement frequency of 1 min^−1^. In this way, the effect of rising gas bubbles on the SL signal was eliminated leading to stable SL signal for a constant volumetric flow rate.

A correlation between the scattered light signal and optical density or bio dry weight, respectively, can be found in the Supplementary information (SI2 *Correlation of scattered light signal, optical density and cell dry weight*).

Dissolved oxygen tension (DOT) and pH were measured online using phosphorescent sensor spots that were attached to the sensor plate (see Fig. 1A). The latter was a transparent polycarbonate (PC) slide (58 mm × 25 mm × 2 mm), which closed the reaction volume in the back of the 3D-µBCR and enabled noninvasive read out of the sensor signals with a four‐channel phase‐shift fluorimeter FireStingO2 (PyroScience, Aachen, Germany). For a higher adhesion of sensor material and PC-slide, circles with a diameter of 300 µm were treated using a CNC milling machine. Sensor polymer solutions were then inkjet-printed onto these spots, as previously described by Lladó Maldonado et al.^11^. Checking the signal intensity of the respective spots ensures adequate layer thickness. These sensors were then positioned to measure the analytes in the fluid phase of the 3D-µBCR. To allow for stable and precise sensor read out, optical fibers (*d*_i_ = 1 mm, I-V2Y 1P, Helukabel GmbH, Hemmingen, Germany) were also integrated into a custom-made mounting by fixing the fibers onto the backside of the 3D-µBCR. Both pH and DOT were continuously measured online (every 10 s unless stated otherwise). Calibration of the oxygen sensor was performed in the appropriate solution or cultivation medium and at temperatures of the subsequent application similar to those report by Nacht et al.^40^. First, the 3D-µBCR was filled with fluid and flushed with nitrogen to determine the phase shift at deoxygenated conditions corresponding to 0% dissolved oxygen saturation. Then, gassing was switched to ambient air and the measured phase shift was estimated as 100% oxygen saturation. The applied pH sensor displayed a dynamic range between pH 4 and 9^41^. The sensors were incubated in 0.9% (w/v) NaCl-solution at room temperature for 48 h, in order to ensure sufficient sensor equilibration. Subsequent multi-point calibration was performed using 150 mM phosphate buffer solutions, transferred under sterile conditions via a 0.2 µm filter (Minisart, Sartorius, Göttingen, Germany) into the 3D-µBCR. Adjusting the relation between KH_2_PO_4_ and K_2_HPO_4_, solutions with different pH (4.5; 6; 6.5; 7; 7.5; 9) were prepared. Prior to each calibration, the buffer solutions were equilibrated at appropriate temperature and their pH was analyzed using an external pH-meter (FiveEasy Plus, Mettler Toledo GmbH, Giessen, Germany).

In addition to the sensors used to take measurements in the fluid phase, relative CO_2_ and O_2_ concentrations were also measured in the off-gas via an infrared based sensor (BlueInOne, BlueSense, Herten, Germany). Four different CO_2_ concentrations were measured for calibration purposes (0.0; 2.5; 5.0; 7.5% (v/v)) in duplicate until a constant sensor signal was achieved but at least for 30 min.

### Hydrodynamic and mass transfer characterization

The mixing time *t*_M_ was analyzed depending on the volumetric flow rate using a colorimetric method. Therefore, the appropriate flow rate was adjusted via the mass flow controller (El-Flow, Bronkhorst High-Tech B.V., Ruurlo, Netherlands) between 0 and 45 mL min^−1^ prior to filling 550 µL deionized (DI) water (Astacus2 LS μS-control, membraPure, Hennigsdorf, Germany) into the 3D-µBCR. Subsequently, 10 µL of an ink solution (4001, Pelikan, Hannover, Germany) was injected into the 3D-µBCR. The mixing process was monitored using a board camera (DFM 72BUC02-ML, The Imaging Source Europe, Bremen, Germany) that was positioned at the back to capture the entire reaction volume through the transparent PC plate. Sufficient illumination was provided by an LED panel (EA LG40X21-A, Electronic assembly GmbH, Gilching, Germany). For each mixing process, a video was captured with 50 fps using the imaging software IC Capture 2.4 (The Imaging Source Europe, Bremen, Germany), which was analyzed using an in-house Python program utilizing OpenCV library for image analysis. To define the actual reaction volume, a region of interest (ROI) was selected. In this ROI, mean grey scale values were calculated for a user defined set of images for every frame and compared to the previous frame using Eq. 3^42^.

3 $$I_{grey} = \frac{1}{{n_{{{\text{pixel}}}} }} \cdot \mathop \sum \limits_{n}^{i = 1} I_{{{\text{grey}},{\text{i}}}}$$

*n*_pixel_ is the number of pixels that is spanned by the ROI. Next, the greyscale values were normalized by an initial (at time point *t*_0_) and final reference frame (at time point *t*_f_), using Eq. 4 to calculate the level of homogeneity.

4 $$I_{normalized} \left( {t_{i} } \right) = \frac{{I_{grey} \left( {t_{i} } \right) - I_{grey} \left( {t_{0} } \right)}}{{I_{grey} \left( {t_{f} } \right) - I_{grey} \left( {t_{0} } \right)}}$$

Mixing time *t*_M_ was defined as the time difference between the initial frame and the frame at corresponding time t* where 95% homogeneity is reached (Eq. 5).

5 $$t_{M} = t^{*} - t_{0} , i_{{{\text{normalized}}}} \left( {t^{*} } \right) \ge 0.95$$

All measurements were performed in triplicates.

The volumetric mass transfer coefficient *k*_L_*a* was determined using the dynamic gassing-out method, where the previously described oxygen sensor spot was used to determine the dissolved oxygen tension. To this end, the 3D-µBCR was filled with 550 µL DI-water (Astacus2 LS μS-control, membraPure, Hennigsdorf, Germany) and heated up to 37 °C. Subsequently, the fluid was gassed with nitrogen to assure deoxygenated conditions. Gassing was then instantly switched to ambient air. To calculate the *k*_L_*a*, the increase of the dissolved oxygen tension up to 40% was used, applying Eq. 6.

6 $$k_{{\text{L}}} a = {\text{ln}}\frac{{c_{{{\text{O}}_{2} }}^{*} - c_{{0,{\text{O}}_{2} }} }}{{c_{{{\text{O}}_{2} }}^{*} - c_{{{\text{O}}_{2} }} }}/t$$

Here, $c_{{{\text{O}}_{2} }}$ is the measured dissolved oxygen concentration, $c_{{O_{2} }}^{*}$ is the dissolved oxygen saturation concentration, and $c_{{0,{\text{O}}_{2} }}$ is the dissolved oxygen concentration at *t* = 0 h ^32,43^.

Bubble size was determined via static imaging of the 3D-µBCR at various volumetric flow rates, using the camera setup previously described in connection with determining *t*_M_. The 3D-µBCR was filled with 550 µL DI-water, after which the flow rate to be examined was adjusted. Subsequently, 1000 images were automatically taken and then analyzed using the image processing software ImageJ (National Institutes of Health, Bethesda, USA). To clearly distinguish between gas bubbles and background, the images were converted into 8-bit greyscale images which allowed the bubbles to be highlighted and then analyzed for their major and minor diameter. The bubbles can be described as a rotation ellipsoid, from which the diameter of a spherical bubble (SB) can be calculated using Eq. 7.

7 $$d_{{{\text{SB}}}} = \sqrt[3]{{8 \cdot h^{2} \cdot z}} = \sqrt[3]{{8 \cdot \left( {\frac{{d_{major} }}{2}} \right)^{2} \cdot \frac{{d_{minor} }}{2}}}$$

Here *h* is the length of the bubble in longitudinal axis, and *z* is the length in the other axis, respectively.

### Computational fluid dynamics

The transient fluid motion is modeled with the Eulerian multiphase (EMP) approach. EMP handles each phase as interpenetrating continua, without resolving the interface between the two phases explicitly. The transport equations for mass and momentum of each phase are solved sharing a mutual pressure field. Due to the Eulerian averaging approach, however, this model does require additional interaction correlations (e.g., drag force, lift force, etc.) between the phases^44^. For the sake of brevity in this paper, the governing equations, interaction correlations, boundary conditions, and solver settings are all provided in the Supplementary information (see section SI3 *Computational Fluid Dynamics*).

In this study, only the drag force was taken into account as an additional force in the source term of the momentum equation. The correlation from Tomiyama et al.^45^ with moderate contamination is used to predict the drag coefficient. Mixing time in the liquid phase is simulated in line with the experimental procedure, but via a virtual tracer (scalar $\phi$)—see details in, e.g., Wehinger et al.^46^. The mixing time is evaluated after a steady-state-like flow regime has developed and is defined as the time required to obtain a 95% level of homogeneity^26^. This state is monitored by the uniformity index (see Supplementary information, SI3 *Computational Fluid Dynamics*). The EMP model is implemented and solved in Simcenter STAR-CCM + 2020.1 by Siemens Digital Industries Software.

### Cultivation of *Saccharomyces cerevisiae* CCOS 538

For validation purposes of the 3D-µBCR system, cultivations with the facultative anaerobic growing yeast *S. cerevisiae* CCOS 538 (ATCC 32,167) were performed. The strain was received from the Culture Collection of Switzerland AG (Waedenswil, Switzerland) and cultivated in a chemically defined medium, as previously described by Krull and Peterat^1^, and Peterat et al.^9^. The initial glucose concentration was 20 g L^−1^ and the pH was set to 4.5 (FiveEasyPlus, Mettler Toledo GmbH, Giessen, Germany). All components were purchased from Carl Roth (Karlsruhe, Germany). Inocula were grown overnight at 30 °C in 250 mL shaking flasks (Schott, Mainz, Germany), containing four baffles, filled with 10% at 180 min^−1^ (shaking diameter 50 mm, ISF1-X, Adolf Kühner AG, Birsfelden, Switzerland). A second culture was inoculated to an OD of 0.3 using the overnight culture and incubated for another 8 h at 30 °C and 180 min^−1^, which was subsequently used to inoculate a third culture to be applied in the 3D-µBCR with a starting OD of 0.3. The cultivation was online monitored using the previously described optical sensors for biomass, DOT and pH. Furthermore, the off-gas was analyzed for CO_2_ and O_2_ content (BlueInOne, BlueSense, Herten, Germany). The OD was additionally determined offline using a spectrometer (Libra S11, Biochrom Ltd., Cambourne, UK).

## Supplementary Information

Supplementary Information

## References

[1] Krull R, Peterat G (2016). Analysis of reaction kinetics during chemostat cultivation of *Saccharomyces cerevisiae* using a multiphase microreactor. Biochem. Eng. J..

[2] Schmitz J, Noll T, Grünberger A (2019). Heterogeneity studies of mammalian cells for bioproduction: From tools to application. Trends Biotechnol..

[3] Marques M, Szita N (2017). Bioprocess microfluidics: Applying microfluidic devices for bioprocessing. Curr. Opin. Chem. Eng..

[4] Hemmerich J, Noack S, Wiechert W, Oldiges M (2018). Microbioreactor systems for accelerated bioprocess development. Biotechnol. J..

[5] Krull R, Lladó Maldonado S, Lorenz T, Demming S, Büttgenbach S, Dietzel A (2016). Microbioreactors in *Microsystems for pharmatechnology*. Manipulation of fluids, particles, droplets, and cells.

[6] El-Ali J, Sorger PK, Jensen KF (2006). Cells on chips. Nature.

[7] Edlich A (2010). Microfluidic reactor for continuous cultivation of *Saccharomyces cerevisiae*. Biotechnol. Prog..

[8] Demming S (2012). Vertical microbubble column—a photonic lab-on-chip for cultivation and online analysis of yeast cell cultures. Biomicrofluidics.

[9] Peterat G (2014). Characterization of oxygen transfer in vertical micro bubble columns for aerobic biotechnological screening processes. Biotechnol. Bioeng..

[10] Lladó Maldonado S (2018). Multiphase microreactors with intensification of oxygen mass transfer rate and mixing performance for bioprocess development. Biochem. Eng. J..

[11] Lladó Maldonado S (2019). A fully, online sensor-equipped, disposable multiphase microbioreactor as a screening platform for biotechnological applications. Biotechnol. Bioeng..

[12] Lladó Maldonado S (2019). Application of a multiphase microreactor chemostat for the determination of reaction kinetics of *Staphylococcus carnosus*. Bioproc. Biosys. Eng..

[13] Preuss JA, Nguyen GN, Berk V, Bahnemann J (2020). Miniaturized free-flow electrophoresis: production, optimization, and application using 3D printing technology. Electrophoresis.

[14] Enders A, Siller IG, Urmann K, Hoffmann MR, Bahnemann J (2018). 3D printed microfluidic mixers—a comparative study on mixing unit performances. Small.

[15] Zhu Y (2018). Propidium monoazide pretreatment on a 3D-printed microfluidic device for efficient PCR determination of ’live versus dead’ microbial cells. Environ. Sci. Water Res. Technol..

[16] Ong LJY (2017). A 3D printed microfluidic perfusion device for multicellular spheroid cultures. Biofabrication.

[17] Lee W (2014). Ultrarapid detection of pathogenic bacteria using a 3D immunomagnetic flow assay. Anal Chem..

[18] Krujatz F (2017). Additive biotech—Chances, challenges, and recent applications of additive manufacturing technologies in biotechnology. N. Biotechnol..

[19] Krujatz F (2016). MicrOLED-photobioreactor: Design and characterization of a milliliter-scale flat-panel-airlift-photobioreactor with optical process monitoring. Algal. Res..

[20] Panjan P, Virtanen V, Sesay AM (2018). Towards microbioprocess control: an inexpensive 3D printed microbioreactor with integrated online real-time glucose monitoring. Analyst.

[21] Cox, C. S. A multi-channel 3D-printed bioreactor for evaluation of growth and production in the microalga *Dunaliella* sp.*,* PhD thesis, The University of Maine, USA (2016).

[22] Arshavsky-Graham S, Enders A, Ackerman S, Bahnemann J, Segal E (2021). 3D-printed microfluidics integrated with optical nanostructured porous aptasensors for protein detection. Microchim. Acta..

[23] Kheradmandnia S, Hashemi-Najafabadi S, Shojaosadati SA, Mousavi SM, Malek Khosravi K (2015). Development of parallel miniature bubble column bioreactors for fermentation process. J. Chem. Technol. Biotechnol..

[24] Gill NK, Appleton M, Baganz F, Lye GJ (2008). Design and characterisation of a miniature stirred bioreactor system for parallel microbial fermentations. Biochem. Eng. J..

[25] Islam RS, Tisi D, Levy MS, Lye GJ (2008). Scale-up of *Escherichia coli* growth and recombinant protein expression conditions from microwell to laboratory and pilot scale based on matched k_L_a. Biotechnol. Bioeng..

[26] Maier U, Büchs J (2001). Characterisation of the gas-liquid mass transfer in shaking bioreactors. Biochem. Eng. J..

[27] Kraume M (2012). Transportvorgänge in der Verfahrenstechnik, 555.

[28] Bothe D, Koebe M, Wielage K, Prüss J, Warnecke HJ, Sommerfeld M (2004). Direct numerical simulation of mass transfer between rising gas bubbles and water. Bubbly flows, heat mass transfer.

[29] Peterat, G. Prozesstechnik und reaktionskinetische Analysen in einem mehrphasigen Mikrobioreaktorsystem*,* in *ibvt-Schriftenreihe* (ed. Krull, R.), Vol. 75, doctoral thesis, Braunschweig University of Technology, Germany (Cuvillier-Verlag, Göttingen, Germany, 2014).

[30] Nienow AW (2013). The physical characterisation of a microscale parallel bioreactor platform with an industrial CHO cell line expressing an IgG4. Biochem. Eng. J..

[31] Alméras E (2016). Scalar mixing in bubbly flows: Experimental investigation and diffusivity modelling. Chem. Eng. Sci..

[32] Kirk TV, Szita N (2013). Oxygen transfer characteristics of miniaturized bioreactor systems. Biotechnol. Bioeng..

[33] Schäpper D, Alam MNHZ, Szita N, Eliasson Lantz A, Gernaey KV (2009). Application of microbioreactors in fermentation process development: A review. Anal. Bioanal. Chem..

[34] Rieger M, Käppeli O, Fiechter A (1983). The role of limited respiration in the incomplete oxidation of glucose by *Saccharomyces cevevisiae*. J. General. Microbiol..

[35] Furukawa K, Heinzle E, Dunn IJ (1983). Influence of oxygen on the growth of *Saccharomyces cerevisiae* in continuous culture. Biotechnol. Bioeng..

[36] Otterstedt K (2004). Switching the mode of metabolism in the yeast *Saccharomyces cerevisiae*. EMBO Rep..

[37] Paalme T, Elken R, Vilu R, Korhola M (1997). Growth efficiency of *Saccharomyces cerevisiae* on glucose/ethanol media with a smooth change in the dilution rate (A-stat). Enzyme Microb. Technol..

[38] Kuhlmann W, Meyer HD, Bellgardt KH, Schügerl K (1984). On-line analysis of yeast growth and alcohol production. J. Biotechnol..

[39] Siller IG (2019). Real-time live-cell imaging technology enables high-throughput screening to verify in vitro biocompatibility of 3D printed materials. Materials.

[40] Nacht B (2015). Integrated catheter system for continuous glucose measurement and simultaneous insulin infusion. Biosens. Bioelectron..

[41] Gruber P, Marques M, Szita N, Mayr T (2017). Integration and application of optical chemical sensors in microbioreactors. Lab Chip.

[42] Frey LJ (2019). Novel electrodynamic oscillation technique enables enhanced mass transfer and mixing for cultivation in micro-bioreactor. Biotechnol. Prog..

[43] Garcia-Ochoa F, Gomez E, Santos VE, Merchuk C (2010). Oxygen uptake rate in microbial processes: an overview. Biochem. Eng. J..

[44] Ishii M, Hibiki T (2011). Thermo-fluid dynamics of two-phase flow.

[45] Tomiyama A, Kataoka I, Zun I, Sakaguchi T (1998). Drag coefficients of single bubbles under normal and micro gravity conditions. *JSME Int*. J. Ser. B Fluids Therm. Eng..

[46] Wehinger GD, Kreitz B, Nagy A, Turek T (2020). Characterization of a modular Temkin reactor with experiments and computational fluid dynamics simulations. Chem. Eng. J..

