# Supplementary material for: 3D-printed micro bubble column reactor with integrated microsensors for biotechnological applications: From design to evaluation
Source: Sci Rep. 2021 Mar 31;11:7276. doi: 10.1038/s41598-021-86654-9 (PMC8012708; doi:10.1038/s41598-021-86654-9)
Supplement: Supplementary file 1 — Supplementary Information [file 41598_2021_86654_MOESM1_ESM.docx]

**Supplementary information**

**3D-printed micro bubble column reactor with integrated microsensors for biotechnological applications: From design to evaluation**

*Lasse Jannis Frey, David Vorländer, Hendrik Ostsieker, Detlev Rasch, Jan-Luca Lohse, Maximilian Breitfeld, Jan-Hendrik Grosch, Gregor D. Wehinger, Janina Bahnemann, Rainer Krull**

SI1 Sensor controlling and read out

Source code for reading out the infrared temperature sensor (MLX90614, Melexis NV, Ypern, Belgium) via microcontroller (ATMega328, Arduino nano, Arduino S.r.l., Nizza, Italy):

#include <Wire.h>

#include <Adafruit_MLX90614.h>

#include <LiquidCrystal_I2C.h>

LiquidCrystal_I2C lcd(0x27,20,4);

Adafruit_MLX90614 mlx = Adafruit_MLX90614();

float data[40];

float offset = 0.98;

float Treal;

void **setup**() {

lcd.begin();

lcd.setCursor(0,0);

lcd.print("LED-Power = ");

lcd.setCursor(0,1);

lcd.print("Object = ");

lcd.setCursor(0,2);

lcd.print("Off-Set = ");

lcd.print(offset);

lcd.print("\337C");

lcd.setCursor(0,3);

lcd.print("Textern = ");

mlx.begin();

Serial.begin(9600);

}

void loop() {

int sensorValue = analogRead(A0);

float voltage = sensorValue / 1023.0*100;

float Tamb = mlx.readAmbientTempC();

float readedsensor = readsensor();

float Tex = readedsensor + offset;

lcd.setCursor(13,0);

lcd.print(voltage);

//lcd.print("%");

//lcd.setCursor(10,0);

//lcd.print(Tamb);
//lcd.print("\337C");

lcd.setCursor(10,1);

lcd.print(readedsensor);

lcd.print("\337C");

lcd.setCursor(10,3);

lcd.print(Tex);

lcd.print("\337C");

//Serial.print("OUT_PV_05_0");

Serial.println(Tex);

}

float readsensor(){

int i;

for (i = 0; i < 40; i = i + 1) {

data[i] = mlx.readObjectTempC();

delay(250);

}

return(average(data, 40));

Serial.println(average(data, 40));

}

float average (float * array, int len)

{

float sum = 0L ;

for (int i = 0 ; i < len ; i++)

sum += array [i] ;

return ((float) sum) / len ;

}

Source code for data transmission between the microcontroller (ATMega328, Arduino nano, Arduino S.r.l., Nizza, Italy) and thermostat written in “Processing”:

import processing.serial.*;

Serial myPort;

Serial myPort2; /

String raw;

String eco;

void **setup**()

{

String portName = Serial.list()[0];

myPort = new Serial(this, portName, 9600);

String portName2 = Serial.list()[1];

myPort2 = new Serial(this, portName2, 9600);

myPort2.write("STOP" + "\r");

delay(5000);

myPort2.write("START" + "\r");

}
void **draw**()

{

if ( myPort.available() > 0)

{ // If data is available,

raw = myPort.readStringUntil(&apos;\n&apos;);

eco = "OUT_PV_05_0" + raw;

}

System.out.println(eco);

myPort2.write(eco + "\r");

delay(1000);

}

SI2 Correlation of scattered light signal, optical density and cell dry weight

To determine the cell dry weight, 18 Corex glasses with a respective volume of 50 mL were cleaned and rinsed thoroughly, to avoid any contamination, and allowed to dry overnight at 80 °C and scaled (CP225D, Sartorius AG, Göttingen, Germany). Subsequently, six Erlenmeyer flasks with a volume of 500 mL were filled with 60 mL of modified Verduyn medium (see section Methods) and inoculated using an overnight culture of *Saccharomyces cerevisiae* CCOS 538. The following cultivation was performed at 30 °C and shaking frequency of 180 min^-1^ (shaking diameter 50 mm). The culture was sampled every 30 mins under sterile conditions and the optical density was determined using a cuvette spectrometer (Libra S11, Biochrom Ltd., Cambourneat, UK) at a wavelength of 600 nm. Additionally, every hour 3×15 mL cultivation broth was withdrew under sterile conditions and transferred to a Corex glass. The filled samples of cultivation broth were centrifuged for 10 min at 6000 min^-1^ and 4 °C (Hercules Multifuge X1R, Thermo Fisher Scientific Inc., Waltham, USA). The supernatant was discarded and the resulting pellet was scaled after 48 h of drying at 80 °C.

To correlate the scattered light signal with optical density, *S. cerevisiae* CCOS 538 was cultivated in modified Verduyn medium, as reported previously, in the µBC-MBR at 30 °C. Over the course of the cultivation, 50 µL of the cell suspension (cultivation broth) were withdrew and the optical density was analyzed offline using a spectrometer (Libra S11, Biochrom Ltd., Cambourneat, UK) (**Figure S1**).


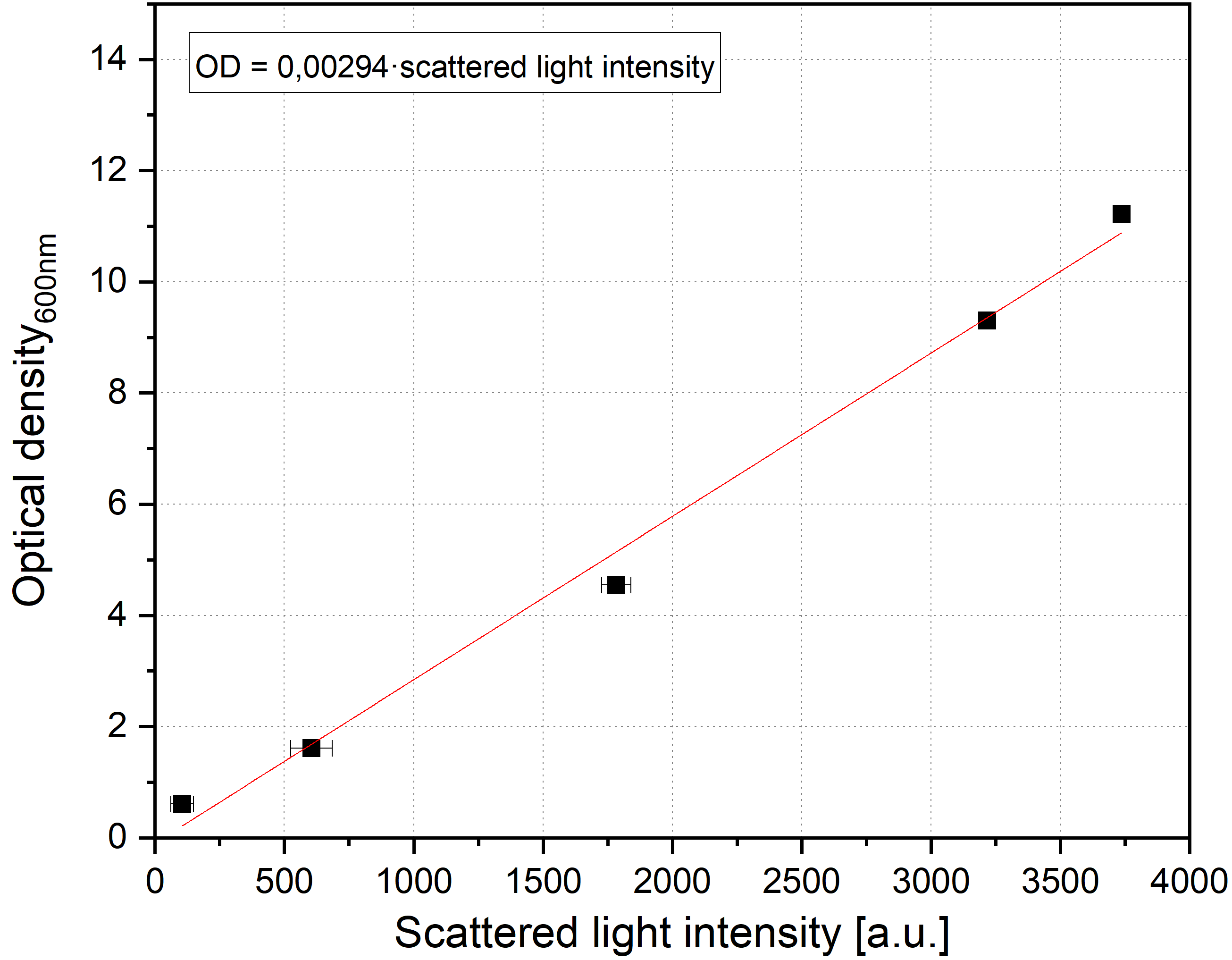

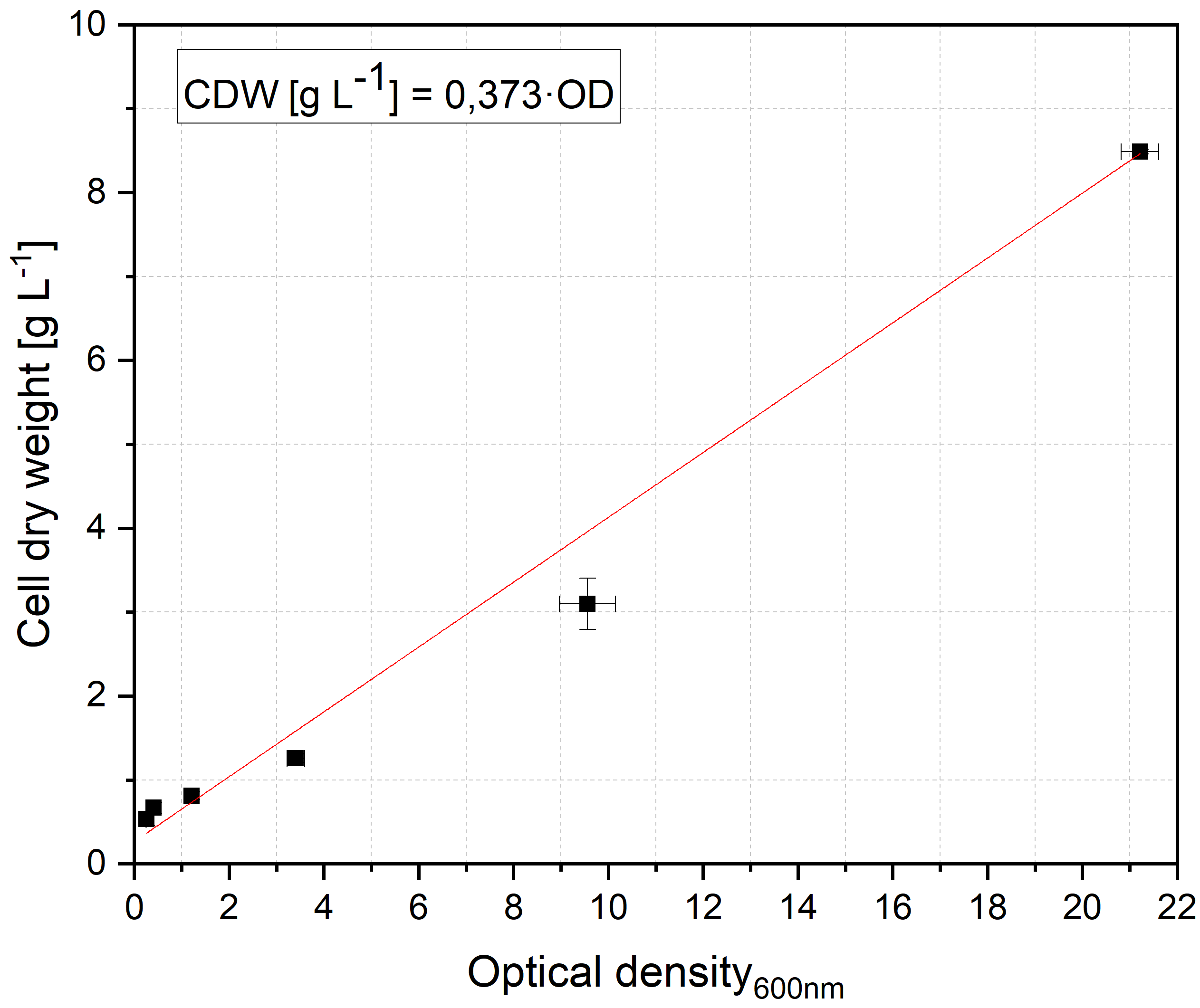


**Figure S1:** Correlation between scattered light intensity and optical density of *S. cerevisiae* CCO 538 measured in the 3D-µBCR (see section Methods) (left). Correlation between optical density and cell dry weight of *S. cerevisiae* CCO 538.

SI3 Computational Fluid Dynamics

In this study, the transient fluid motion of the coupled liquid and gas phase is modeled with the Eulerian multiphase (EMP) approach. Here, the volume fraction *α* of phase *i* is defined as (**Equation S1**):

| $\alpha_{i}=\frac{V_{i}}{V}$ | (S1) |
| --- | --- |

where $V_{i}$ is the share of the volume occupied by phase $i.$ The volume fractions of all phases (**Equation S2**) sum up to unity $1=\sum_{i=0}^{n} V_{i}$. (S2)
The conservation of mass of phase *i* reads (**Equation S3**):

| $\frac{\partial}{\partial t}\int_{\tilde{V}} \alpha_{i}\rho_{i}dV+\oint_{A} \alpha_{i}\rho_{i}\mathbf{v}_{i}\cdot d\mathbf{a}=0$ | (S3) |
| --- | --- |

with subscript *i* indicating the individual phase and *ρ* being the density and *v* the velocity vector.

The conservation of momentum of phase *i* reads (**Equation S4**):

| $\frac{\partial}{\partial t}\int_{\tilde{V}} \alpha_{i}\rho_{i}\mathbf{v}_{i}dV+\oint_{A} \alpha_{i}\rho_{i}\mathbf{v}_{i}\bigotimes\mathbf{v}_{i}\cdot d\mathbf{a}=-\int_{\tilde{V}} \alpha_{i}\nabla pdV+\int_{\tilde{V}} \alpha_{i}\rho_{i}\mathbf{g}dV+\oint_{A} \alpha_{i}\mathbf{T}_{i}\cdot d\mathbf{a+}\int_{\tilde{V}} \mathbf{F}dV$ | (S4) |
| --- | --- |

where $p$ is pressure, $\mathbf{g}$ is the gravity vector, $\mathbf{T}_{i}$ is the molecular stress, $\mathbf{F}$ are additional forces. In this study, only the drag force $\mathbf{F}_{D}$ is taken into account, i.e., $\mathbf{F=}\mathbf{F}_{D}$. Other typical forces in the EMP framework, like virtual mass force, lift force, turbulent dispersion force or wall lubrication force are omitted, since they do not apply to the described flow conditions, especially gas flow rate, and reactor dimensions.

The drag force is calculated as a function of the drag coefficient *C*_D_, which can be approximated with several different correlations^S1^. The drag force reads (**Equation S5**)^S2^:

| $\mathbf{F}_{D}=-\frac{3}{4d_{b}}C_{D}\rho_{c}\alpha_{d}\left\vert\mathbf{v}_{c}-\mathbf{v}_{d} \right\vert\left( \mathbf{v}_{c}-\mathbf{v}_{d} \right)$ | (S5) |
| --- | --- |

where $d_{b}$ is the bubble diameter, and $C_{D}$ is the drag coefficient. In this study, the correlation from Tomiyama et al.^44^ with moderate contamination is used (**Equation S6**):

| $C_{D}=max\left( min\left( \frac{24}{Re}\left( 1+0.15{Re}^{0.687} \right),\frac{72}{Re} \right),\frac{8Eo}{3\left( Eo+4 \right)} \right)$ | (S6) |
| --- | --- |

where Re is the Reynolds number (**Equation S7**) and *E*_o_ is the Eötvös number (**Equation S8**), respectively.

| $Re=\frac{\rho_{c}\left\vert\mathbf{v}_{c}-\mathbf{v}_{d} \right\vert l}{\mu_{c}}$ | (S7) |
| --- | --- |
| $Eo=\frac{\left\vert\rho_{c}-\rho_{d} \right\vert l^{2}}{\sigma}$ | (S8) |

with $l$ as the bubble size or interaction length scale, $\sigma$ as the surface tension, $\mu$ as the dynamic viscosity.

In an 8 mm^3^ cube in the upper part of the column, the scalar $\phi$ is introduced to the liquid phase and transported by convection only (**Equation S9**):

| $\frac{\partial}{\partial t}\int_{\tilde{V}} \rho_{c}\phi dV+\oint_{A} \rho_{c}\phi\mathbf{v}_{i}\cdot d\mathbf{a}=\oint_{A} \left( \frac{\mu}{\rho Sc}+\frac{\mu_{t}}{{\rho Sc}_{t}} \right)\nabla\phi\cdot d\mathbf{a}$ | (S9) |
| --- | --- |

where the second term on the left side represents the convective transport and the right-hand side of the equation is transport by diffusion. $\mu$ is the viscosity and $\rho$ the density of the fluid and $Sc$ is the Schmidt number with subscript $t$ representing the turbulent portion. The molecular and turbulent Schmidt numbers are set to 0.7.

The mixing time in the CFD model is calculated via a virtual tracer experiment and monitored with the uniformity index^S3^. On the central plane section of the calculation domain, this value is defined as (**Equation S10**):

| $Uniformity index of \phi=1-\frac{\sum_{i} \left\vert\phi_{f}-\bar{\phi} \right\vert A_{f}}{2\left\vert\bar{\phi} \right\vert\sum_{i} A_{f}}$ | (S10) |
| --- | --- |

where $\bar{\phi}$ is the surface average of scalar $\phi$, $\phi_{f}$ is the face value and $A_{f}$ is the face area. This procedure is in line with the experimental colorimetric method with dynamic imaging.

The computational domain consists of approximately 30,000 polyhedral cells, based on a mesh dependence study (not shown here). A finer mesh resolution was chosen at the top of the initial fluid phase see **Figure S2**.


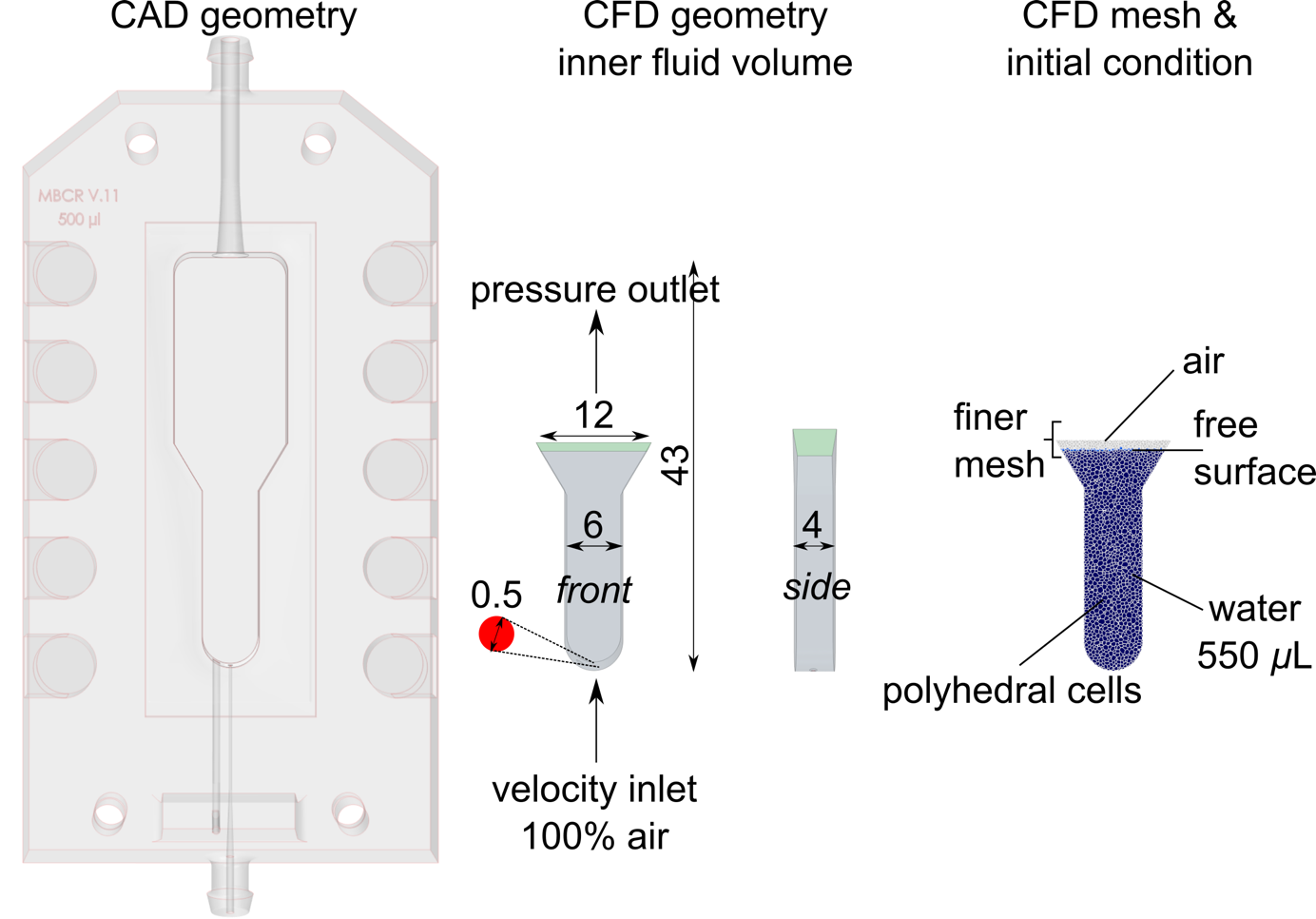


**Figure S2:** Numerical setup: (left) CAD geometry (right) CFD geometry, inner fluid volume, and computational mesh incl. dimensions in mm, boundary and initial conditions.

**Table S1** summarizes the applied fluid properties, model parameters, boundary conditions, and solver settings of the CFD simulations. The EMP model is implemented and solved in Simcenter STAR-CCM+ 2020.1 by Siemens Digital Industries Software.

**Table S1**: Properties and boundary conditions CFD simulations.

| **Fluid properties** | |
| --- | --- |
| Water density / kg m^-3^ | 997.561 |
| Air density / kg m^-3^ | 1.18415 |
| Water dynamic viscosity / Pa s | 8.8871·10^-4^ |
| Air dynamic viscosity / Pa s | 1.85508·10^-5^ |
| **Mesh Properties for Simcenter STAR-CCM+ mesher** | |
| Base size | 0.25 mm |
| Target surface size | 100% |
| Minimum surface size | 2.0% |
| Refinement size in region $\pm$1.25 mm around free surface | 75% |
| **EMP model parameters** | |
| Convection scheme for flow | 2^nd^ order |
| Convection scheme for volume fraction | 1^st^ order |
| Temporal discretization | 1^st^ order |
| Continuous phase | water |
| Dispersed phase | air |
| Drag coefficient model | Tomiyama et al.^44^ with moderate contamination |
| Interaction area density | Symmetric |
| Interaction length scale | 3 mm |
| Surface tension / N m^-1^ | 0.072 |
| Pressure / Pa | 101325.0 |
| Volume flow gas inlet / ml min^-1^ | 3 / 5/ 10 / 20 / 35 |
| Time step / s | Initial time step: $5\cdot{10}^{-4}$ s ; Adaptive with ${CFL}_{max}=50$ and $\bar{CFL}=0.75$ |
| Velocity URF^*^ implicit/explicit | 0.1 / 0.5 |
| Pressure URF^*^ | 0.1 |
| Volume fraction URF* implicit/explicit | 0.2 / 0.5 |
| Turbulence URF* | 0.2 |
| Inner iterations | 15-20 |
| Schmidt number molecular and turbulent (for passive scalar transport) | 0.7 |
| ^*^URF: under-relaxation factor |  |

Supplementary References

S1 Pourtousi, M., Sahu, J. N., Ganesan, P. Effect of interfacial forces and turbulence models on predicting flow pattern inside the bubble column. *Chem. Eng. Process.* **75**, 38-47 (2014).

S2 Clift, R., Grace, J. R., Weber, M. E. Bubbles, drops, and particles (Academic Press, New York, USA 1978).

S3 Weltens, H., Bressler, H., Terres, F., Neumaier, H., Rammoser, D. Optimisation of catalytic converter gas flow distribution by CFD prediction (No. 930780) (SAE Technical Paper 1993).
